# Congenital Agenesis of the Internal Jugular Vein: An Extremely Rare Anomaly

**DOI:** 10.1155/2015/637067

**Published:** 2015-03-03

**Authors:** Oguz Kayiran, Caglar Calli, Abdulkadir Emre, Fatih Kemal Soy

**Affiliations:** ^1^Department of Plastic and Reconstructive Surgery, Izmir University, 35000 Izmir, Turkey; ^2^Department of Head and Neck Surgery, Ekol ENT Hospital, 35000 Izmir, Turkey; ^3^Department of Head and Neck Surgery, Ataturk Training and Research Hospital, 35000 Izmir, Turkey

## Abstract

Vascular anomalies of major venous vessels are rarely seen. Moreover, congenital absence of internal jugular vein is extremely uncommon. In our case, a female patient presented with primary unknown left cervical mass. Cervical ultrasonography demonstrated absence of right internal jugular vein. In addition, computed tomography and dynamic magnetic resonance imaging scans confirmed this diagnosis. Compensatory left internal jugular vein enlargement mimicked sort of cervical mass. Venous magnetic resonance imaging images revealed the absence of right internal jugular vein with compensatory left internal jugular vein dominance. In the literature, the agenesis of IJV was mentioned in a case with concomitant multiple problems. Here, an asymptomatic case is reported with an incident diagnosis. No interventions were planned upon the patient's request. It should be kept in mind that any kind of anomalies can be seen during venous access and neck surgery.

## 1. Introduction

Vascular anomalies are divided into two groups as vascular tumors and vascular malformations according to International Society for the Study of Vascular Anomalies (ISSVA) (Table 1) [1–3]. Vascular tumors consist of hemangioma, hemangioendothelioma, and angiosarcoma. Vascular malformations are comprised of abnormally formed channels that are lined by quiescent endothelium. They are categorized according to the predominant channel type and rheological characteristics as slow-flow (capillary, venous, and lymphatic) and fast-flow (arterial, combined). Developmental venous anomalies in general population are seen in about 0.05% to 0.25%. However, rates up to 20% have been reported [4].

## 2. Case Report

**Figure 1:**
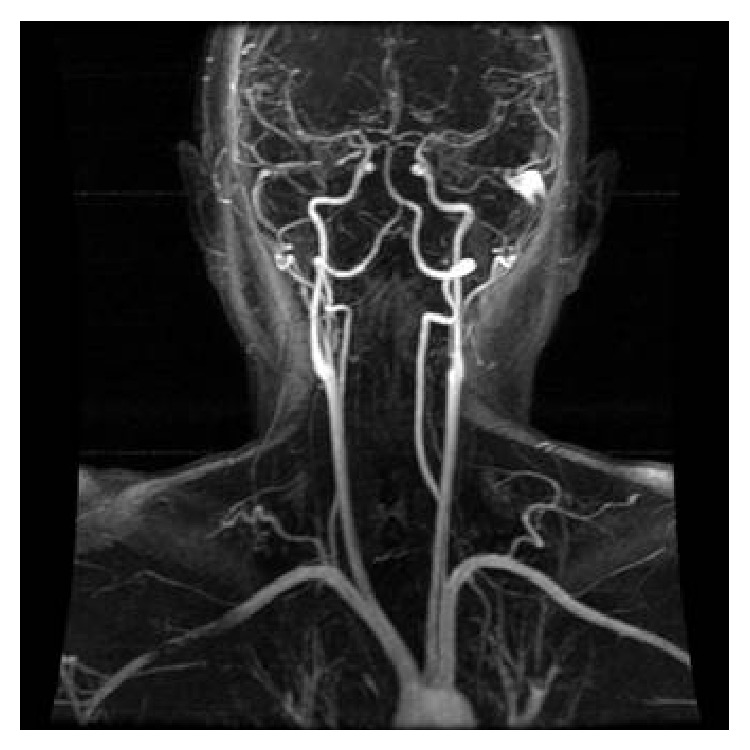
Sagittal view of arterial system (dynamic magnetic resonance image).

**Figure 2:**
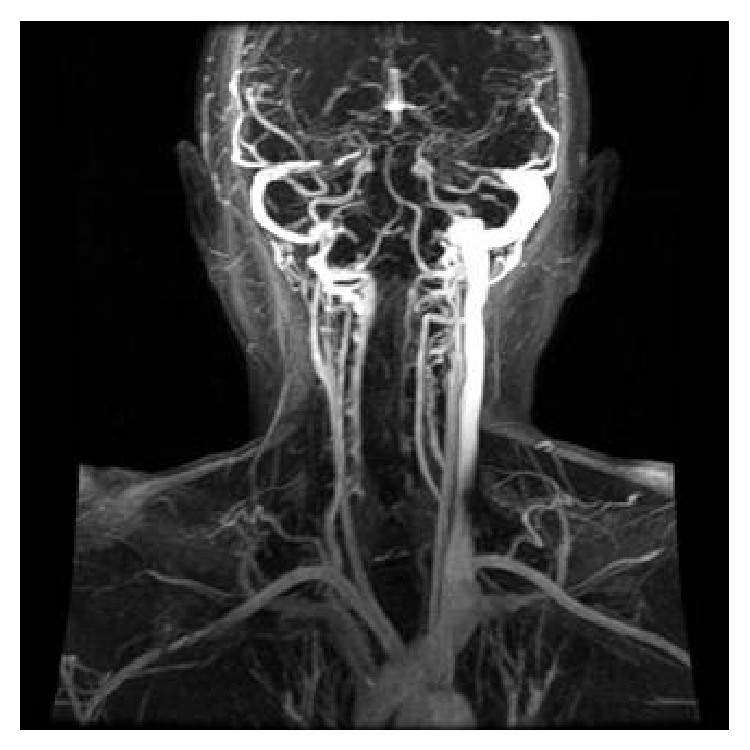
Sagittal view of venous system (dynamic magnetic resonance image). Please note that right filling of IJV with the radiopaque contrast is absent, markedly, from the bifurcation. Moreover, left IJV is seen thickened.

**Figure 3:**
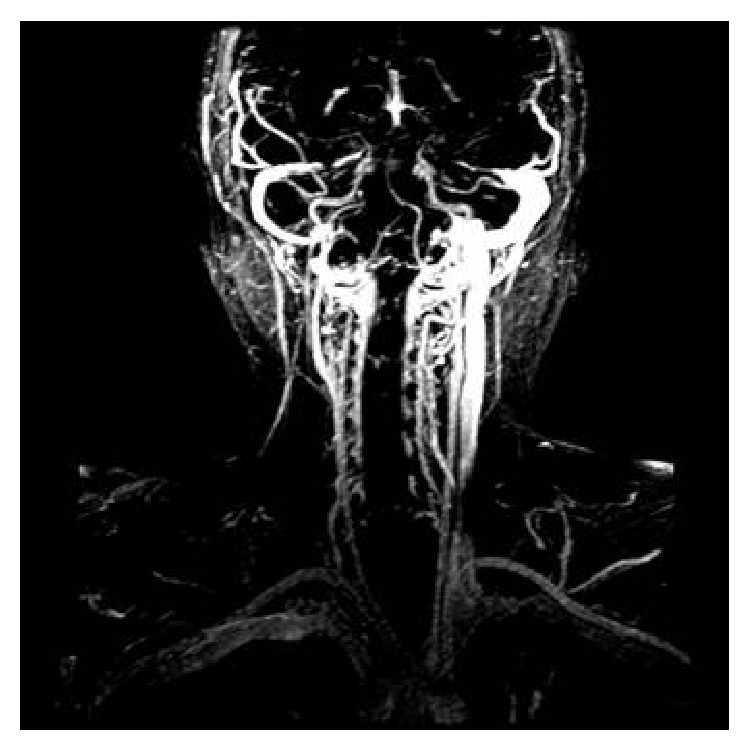
Sagittal view of collateral system (dynamic magnetic resonance image).

17-year-old female patient was referred to Izmir Ataturk Training and Research Hospital Ear, Nose and Throat Clinic, with a painless mass on her left side of the neck existing since childhood. The examination of the head and neck revealed a suspicious mass without color and heat changes at left midjugular region. The physical examination was uneventful. Ultrasonography did not show any mass in the region; however, CT demonstrated absence of right IJV. Doppler USG and dynamic MRI clearly exhibited the same abnormality (Figures 1, 2, and 3). This observation depended on a developmental agenesis rather than a thrombus.

The patient was informed with the situation and no further treatment was planned upon her request. Follow-up periods continue annually within different facilities concomitantly.

## 3. Discussion

Vascular anomalies result from embryological developmental deformities. Hemangiomas regress in time while vascular malformations occasionally progress and remain lifetime. Although vascular malformations exist at birth, they may not be detected until adolescence or adulthood [3]. Venous malformations are mostly asymptomatic and are located at head and neck region, frequently [5]. The vascular anomalies in the head and neck region have tendency to coexist with intracranial venous malformations [4].

IJV runs downwards through the neck within the carotid sheath and behind the sternal end of the clavicle. It then unites within the subclavian vein to form the brachiocephalic vein, which enters the thorax to join vena cava superior.

The information of anatomical variations of IJV is clinically essential for venous applications. In intensive care units, central catheters are frequently applied. Anomalies of IJV may preclude the practitioner resulting in serious complications. The surgeon should be familiar with the anatomy and possible variations of IJV in order to avoid complications. In a study of 93 cadaveric dissections, Asouhidou et al. found that 3 of these dissections showed narrowing of IJV (<6 mm) while the external jugular veins were over normal limits [6]. Duplication of IJV is frequently encountered [7]. Prades et al. showed IJV duplications in 3 of 750 patients during unilateral neck dissections and detected the course of accessory nerve passing through duplicated internal jugular veins [8].

Spontaneous thrombosis of IJV is a very rare condition. In a study of 200 patients whose IJVs were catheterized and examined by two-dimensional ultrasonography, Denys and Uretsky identified nonvisualization of IJV in 5 (2.5%) patients resulting from the thrombosis [9]. The thrombosis of IJV is mostly caused by repeating intravenous injections, drug abuse, polycythaemia, and hypercoagulability, following neck surgery like neck dissection, radiotherapy, deep neck infections, oropharyngeal infections, Lemierre's syndrome, and cervical trauma. Cervical inflammation, mass, or sepsis may also coexist with the thrombosis of IJV [10–12]. Our patient did not have any of the abovementioned conditions and thrombosis antecedently. The ultrasonography, CT, and magnetic resonance imaging did not reveal any thrombosis and showed congenital agenesis of the IJV.

In the literature, one case with the absence of IJV was depicted who also had additional problems [13]. However, further diagnostic analysis and dynamic proofs were not elucidated in this study. In addition, agenesis of both IJVs (right and left) and left external jugular vein has been reported in a cat [14].

Herein, we reported an asymptomatic case of lack of right IJV with compensatory left IJV thickening which was diagnosed accidently. We believe the practitioner should always be aware of possible head and neck anomalies and variations during neck surgeries and vascular interventions.

## Figures and Tables

**Table 1:** ISSVA classification of vascular anomalies.

Tumors	Malformations
Hemangioma Hemangioendothelioma Angiosarcoma Miscellaneous	Slow-flow
	Capillary
	Lymphatic
	Venous
	Fast-flow
	Arterial
	Combined
